# Duchenne Muscular Dystrophy (DMD) Protein-Protein Interaction Mapping

**Published:** 2017

**Authors:** Mostafa REZAEI TAVIRANI, Farshad OkHOVATIAN, Mona ZAMANIAN AZODI, Majid REZAEI TAVIRANI

**Affiliations:** 1 Proteomics Research Center, Shahid Beheshti University of Medical Sciences, Tehran, Iran.; 2 Physiotherapy Research Centre, School of Rehabilitation, Shahid Beheshti University of Medical Sciences, Tehran, Iran.; 3 Faculty of Medicine, Iran University of Medical Sciences, Tehran, Iran.

**Keywords:** Duchenne muscular dystrophy (DMD), Protein-protein interaction network, Gene ontology, Hub-bottlenecks, Biomarker panel

## Abstract

**Objective:**

Duchenne muscular dystrophy (DMD) is one of the mortal diseases, subjected to study in terms of molecular investigation. In this study, the protein interaction map of this muscle-wasting condition was generated to gain a better knowledge of interactome profile of DMD.

**Materials & Methods:**

Applying Cytoscape and String Database, the protein-protein interaction network was constructed and the gene ontology of the constructed network was analyzed for biological process, molecular function, and cellular component annotations.

**Results:**

Among 100 proteins related to DMD, dystrophin, utrophin, caveolin 3, and myogenic differentiation 1 play key roles in DMD network. In addition, the gene ontology analysis showed that regulation processes, kinase activity, and sarcoplasmic reticulum were the highlighted biological processes, molecular function, and cell component enrichments respectively for the proteins related to DMD.

**Conclusion:**

The central proteins and the enriched ontologies can be suggested as possible prominent agents in DMD; however, the validation studies may be required.

## Introduction

Duchenne muscular dystrophy (DMD) is a severe muscle degradation condition that consequent in death of the patient. This inheritable disease is common in males with the prevalence of 1 out of 3500 (1). A functional neural deficit in this disease is reported. However, there are some ethical problems for investigation on the sick boys; EEG abnormality is reported for the number of the patients (2)**. **There is not yet a suitable treatment for DMD (3). 

There are some molecular studies for DMD (4, 5). One of the main extensively changed expressed molecules in this disease is known as dystrophin protein that the lack of its gene expression is linked to the disease development (6). This protein can cause vast amount of changes in morphology and function of skeletal muscle tissue (5). Therefore, it accounts as putative biomarker for DMD. Beside important role of dystrophin, there are other introduced proteins for DMD, mostly conducted by proteomic approaches (7, 8). 

Understanding other related proteins can help for better understanding and managing the disease. In addition to the role of these proteins, the interaction pattern of them can add more information about their contribution in DMD (9). Consequently, protein interaction networks provide information related to systematic communication scale of different kinds of diseases. Proteins that are dysfunctional in a specific disease condition are in complex interactions with each other. Dysregulation of each of these components can induce modification of the interaction of the whole interactome and changes the functions of other proteins. These disruptions of the whole system are responsible for the abnormal phenotype such as diseases (10). Some modification can be referred to the proteins that are essential for an interacted system (11). These proteins are known as central components that their dysfunctions lead to massive changes in the interaction profile (10). Therefore, detecting topological features can be prominent for disease analysis in terms of molecular origin (9). Here for obtaining a better knowledge about molecular mechanisms of DMD, the protein interactome and gene ontology analysis of disease was conducted usingf Cytoscape and DAVID software, respectively. 

## Materials and Methods

Determination of related proteins to a certain disease can be handled through different sources. One of the important sources is Cytoscape 3.4. This common software is free and is compatible with different sources. Many applications are available via Cytoscape and can provide further information for the mapped network. One of the famous interaction sources is String Database (DB). It is accessible through Cytoscape software. Cytoscape can provide different interaction data from different sources. One of them, as mentioned earlier, is the String DB (http://string-db.org/) (12, 13). It has three options for retrieving interaction data including PubMed, disease, and proteins queries. 

Protein interaction map was constructed according to String Database criteria. Here, the disease query was used for extracting 100 top related proteins to DMD. The used cut off for interaction evidence was set at 0.5. This score is a combined score from different sources. The disease score shows the relation of the disease and the obtained proteins. String DB provides interaction information from different sources. Furthermore, for network centrality analysis, Network Analyzer can be used. Here, the examined centrality parameters are degree and betweenness centrality. The nodes with highest degree (connections) are known as hub nodes and similarly, the nodes based on a function of larger number of shortest paths passing through them are called bottlenecks (14). These elements are crucial for the disease onset and progress (15). 

Gene ontology analysis of the whole components of the network was done by the application of The Database for Annotation, Visualization and Integrated Discovery (DAVID) Bioinformatics 6.8 (http://david.niaid.nih.gov). The ontology analysis was based on biological process (BP), cellular component (CC), and molecular function (MF). The associated proteins were clustered for each of these annotations. In each cluster, similar enrichments are included (16). DAVID uses kappa statistics to group the terms. 

## Results

**Fig 1:**
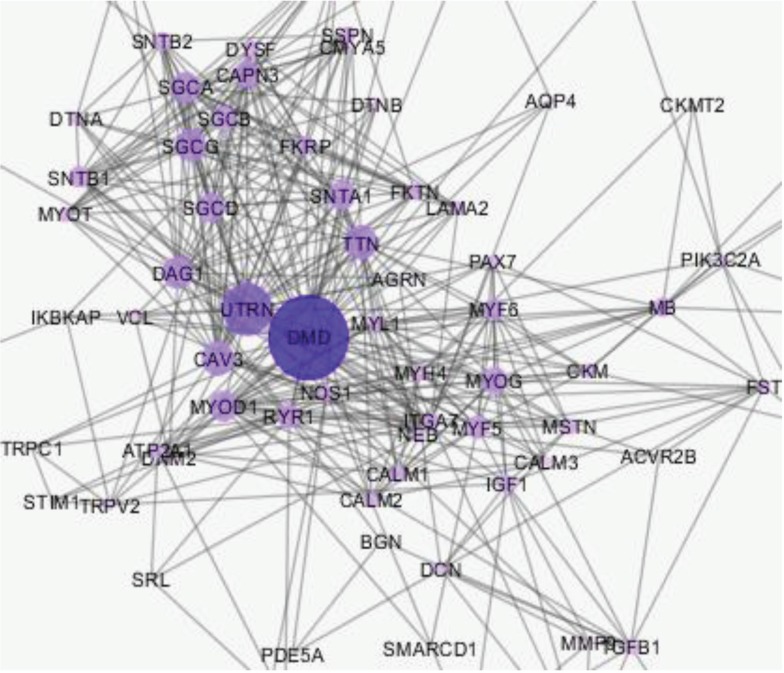
The protein-protein interaction mapping of DMD after analysis with network analyzer, as the nodes get bigger, the degree increases. The network (main connected component) consists of 82 nodes and 395 edges

The protein network was constructed from 100 nodes including 18 isolated nodes and 1 main connected component. The network (main connected component) contains 82 nodes with different disease score relations and 395 edges. The highest disease combined score [4] was assigned for dystrophin protein. This protein also showed the highest value in degree (Figure 1). 

The centrality analysis of the network components based on selection of top 5% of the hub nodes and cut off 0.05 for betweenness revealed that 4 proteins including dystrophin, utrophin, caveolin-3 and myogenic differentiation-1 are hub-bottleneck proteins (Table 1). Since connection between the determined crucial nodes provides informative data, these findings are tabulated in Table 2.

**Table 1:** The list of hub-bottleneck nodes of DMD PPI network based on degree and betweenness centrality analysis

**Gene name**	**Description**	**Degree**	**BC**	**Disease score**
DMD	Dystrophin	40	0.4	4
UTRN	Utrophin	28	0.1	3.9
CAV3	Caveolin 3	19	0.05	2
MYOD1	Myogenic differentiation 1	19	0.1	2.3

**Table 2:** Interactions properties between the determined hub-bottleneck nodes**. **The combined score was set to 0.5. There is no interaction between CAV3 and MYOD1 (16)

**Interactions**	**Combined Score**
CAV3 (pp) DMD	0.992
DMD (pp) UTRN	0.964
MYOD1 (pp) DMD	0.815
CAV3 (pp) UTRN	0.699
MYOD1 (pp)UTRN	0.724

DAVID Bioinformatics Resources 6.8 (16), applied for functional annotation of the all contributed proteins in DMD network including biological process (BP), cell component (CC), and molecular function (MF) (Table 3,4). Each of abbreviations refers to the certain operation for example “RT” (related term) refers to the terms related to the main introduced term in the same row. The accepted *P*-value is 0.1 and the terms are sorted base on *P*-value amounts. Enrichment score corresponds to the important role of the introduced terms in the cluster.

**Table 3:** Clustering annotation of the contributing elements in DMD network based on BP analysis. *Benjamini*–Hochberg is the correction method for *P* value

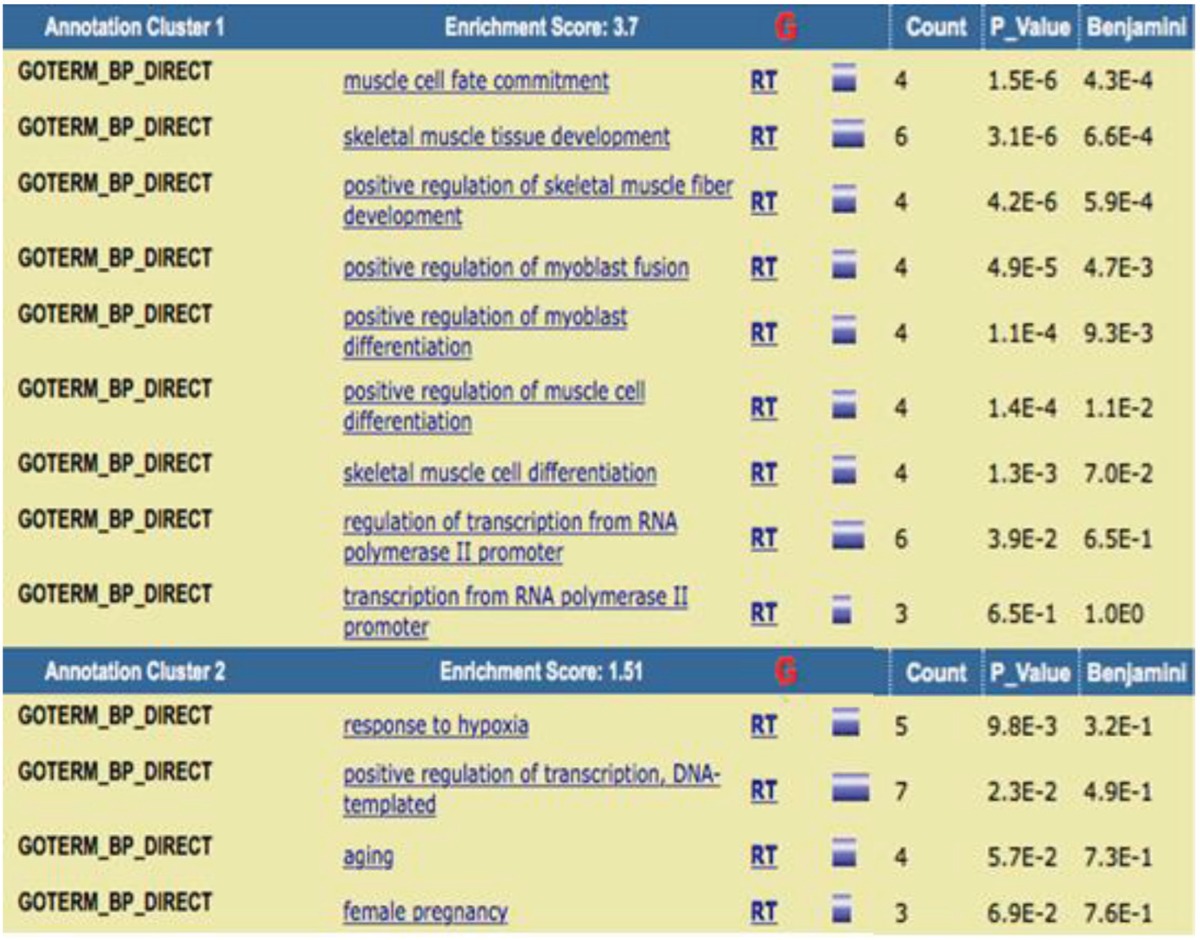

**Table 4:** Clustering annotation of the contributing elements in DMD network based on CC analysis. *Benjamini*–Hochberg is the correction method for *P*-value

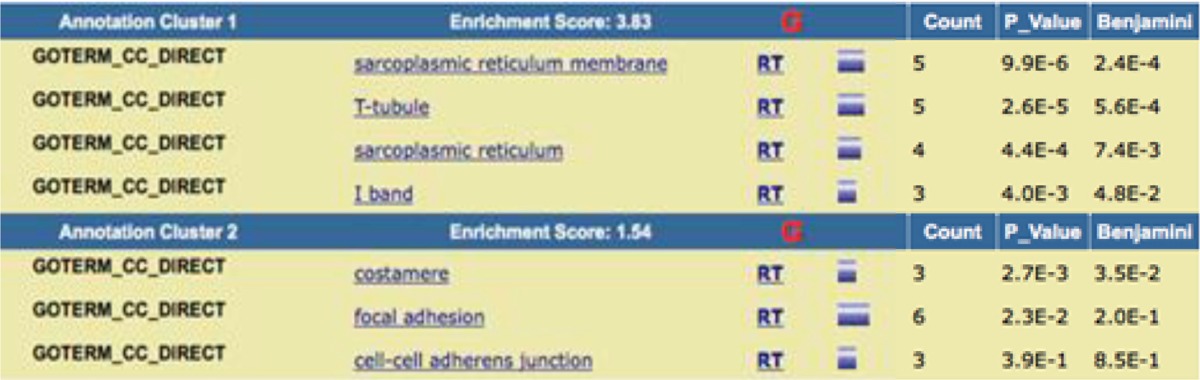

**Table 5:** Clustering annotation of the contributing elements in DMD network based on MF analysis. *Benjamini*–Hochberg is the correction method for *P*-value

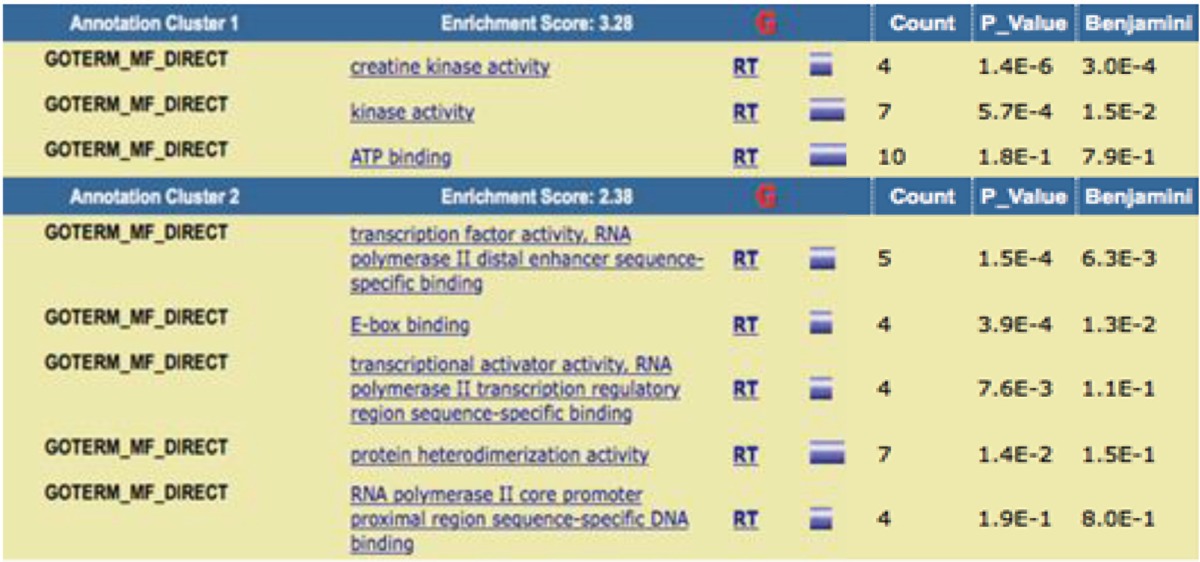

## Discussion

Protein interaction mapping as a powerful analytical method is attracted a great attention of scientists in the field of molecular analysis of diseases (17). One of the recently introduced disciplines is network medicine that investigates about network topological features of a specific disease (18). The information which introduces numbers of essential proteins in terms of interactions can be crucial in disease progression (19). These proteins can be considered as biomarkers that by validation tests would be introduced for clinical approaches. 

The aim of this study was to provide a preliminary knowledge of these key components for more in depth comprehension of Duchenne muscular dystrophy related mechanisms and further investigation in this field. Consequently, a network of all 100 top reported proteins for this disease was constructed and analyzed based on centrality and gene ontology. The associated proteins for this disease were obtained from String source and this source assigned disease score relation based on differently combined evidence. Only 82 genes included in the network and the numbers of 18 genes remained isolated. Each node is connected to the about 5 neighbor nodes. However, due to heterogeneity of the network, the number of the edges for the nodes is different. The centrality analysis of the illustrated network differentiated the nodes and the important genes were highlighted. The important proteins are visible as the largest nodes in the network. Network Analyzer is the applied plug-in for this analysis, provide quantitative information. We choose the most ranked proteins according to the highest degree and largest betweenness centrality. The proteins that showed both characteristics are listed in Table 1. They are known as hub-bottleneck proteins (18). Hub implies on high degree and equally, bottlenecks denote large BC values (20). Therefore, these four proteins including dystrophin, utrophin, caveolin 3, and myogenic differentiation-1 are the key agents of the DMD network. 

Dystrophin as the putative biomarker of DMD (21), also express high centrality values in our network. Attributing top score to dystrophin in our research is a potent validation for the constructed and analyzed PPI network related to DMD. This protein is the top rated node for the integrity of the network. About 10% of all edges are linked to this protein connected to the 50% of all nodes of the network. Dystrophin is characterized by top scores of degree; betweenness and disease score (Table 1). Other key proteins are also important and their role needs more evaluation in the disease. In fact, their involvement with different expression changes has been reported extensively for DMD (3, 22, 23). 

Utrophin the second key protein in our analysis possess near scores to dystrophin in compare to the other two proteins. Its disease score is equal to Utrophin so it is related to DMD strongly. For more resolution, mutual interaction between the crucial proteins as a useful tool was done. In Table 2, the linkage of theses essential proteins together is tabulated. The interactions scores show that the proteins have high degree of interactions between each other. However, only CAV3 and MYOD1 have no connections. The introduced central proteins construct an integrated network related to DMD. Combined score value for the paired genes is higher if DMD is paired. On the other hand, MYOD1 reduces this score. This finding confirms the order of the sorted genes. 

Moreover, to identify the role of these linked proteins to the disease, the gene ontology analysis was used. All the extracted proteins [82] were under consideration of this purpose as they show some association to the disease. Accordingly, DAVID Bioinformatics clustered these agents with the respect to query annotations. The ontology aspects include BP, CC, and MF. The first implemented ontology is biological process of the DMD relevant proteins. In each enrichment analysis, two important clusters are introduced. The first one is the significant cluster and the related terms in this cluster may play more roles that are important in DMD. The main terms in cluster 1 are the biological processes are most active in regulatory processes. Muscle cell fate commitment is the most significant term with the contribution of 4 of the proteins in the network. Skeletal muscle tissue development, positive regulation of skeletal muscle fiber development, positive regulation of myoblast fusion, positive regulation of myoblast differentiation, positive regulation of muscle cell differentiation, skeletal muscle cell differentiation is the other terms related to the involved proteins in DMD. In cell component analysis, the sarcoplasmic reticulum is the proposed resident for the most of the DMD proteins, in which sarcoplasmic reticulum membrane is the most significant part. T tubule and I band are the other two components determined as related parts to the DMD. DAVID functional annotation for MF in Table 5 showed that kinase activity is the highlighted function of the DMD network and creatine kinase activity is the most highlighted one. ATP binding is the other molecular function attributed to the analyzed proteins of DMD PPI network. Investigation about roles of dystrophin (24), utrophin (25), caveolin-3 (26), and myogenic differentiation-1 (27) indicates that relationship between cluster-1 and DMD is highlighted; terms of cluster-1 play roles that are more important in DMD relative to cluster-2.

**In Conclusion, **dystrophin, utrophin, caveolin 3, and myogenic differentiation-1 are introduced as biomarker panel for DMD onset and development. However, the first two proteins play crucial roles. More investigation in the field can lead to validation of the elements of this panel for diagnostic and therapeutic proposes. 
